# Very Severe and Refractory Noninfectious Cystitis in Patients with Systemic Lupus Erythematosus: Potential Role of Rituximab Therapy

**DOI:** 10.1155/2021/6610111

**Published:** 2021-02-27

**Authors:** Vanessa Ocampo-Piraquive, Inés Mondragón-Lenis, Juan G. De los Rios, Carlos A. Cañas

**Affiliations:** ^1^Unit of Rheumatology, Fundación Valle Del Lili, Universidad Icesi, Cali, Colombia; ^2^Department of Internal Medicine, Fundación Valle Del Lili, Universidad Icesi, Cali, Colombia; ^3^Department of Urology, Fundación Valle Del Lili, Universidad Icesi, Cali, Colombia

## Abstract

Systemic lupus erythematosus (SLE) is a chronic inflammatory autoimmune disease with various clinical manifestations, including, rarely, a form of interstitial cystitis (lupus cystitis, LC). LC can be asymptomatic and usually has discrete symptoms that improve with conventional therapies available for SLE and/or typical interstitial cystitis. A very severe and refractory form is rarely described. In this study, we present four patients with SLE and a very severe form of noninfectious cystitis refractory to the different forms of treatment described. The clinical descriptions of the cases, demographic factors, manifestations associated with SLE, and clinical and paraclinical manifestations related to cystitis, treatments, and outcomes are provided. A proposal for the pathogenesis of this condition is based on the common findings of these patients, including the fact that three were in SLE remission and all four receiving rituximab as induction and/or maintenance therapy.

## 1. Introduction

Systemic lupus erythematosus (SLE) is a chronic inflammatory autoimmune disease with various clinical manifestations [1]. One form of interstitial cystitis has been described in these patients since the first report by Fister GM in 1938 [2] and Shipton in 1965 [3] and finally characterized and termed “lupus cystitis” (LC) by Orth et al. in 1983 [4]. LC is a rare manifestation of SLE, with an incidence of 0.5–2.3% [5], and descriptions of LC are based solely on case reports. The reported cases come predominantly from East Asia; the highest incidence has been reported in Korea (2.3% of cases) [6]. In China, incidences of 0.5% and 0.6% [7, 8] have been reported. LC may be the initial presentation of SLE and is predominantly present in women [2, 9].

LC clinically manifests as a variable presence of low irritative and obstructive urinary symptoms, the most common being suprapubic pain, incontinence, polyuria, dysuria, and nocturia; urinalysis may reveal leukocyturia and/or microscopic haematuria with negative urine cultures [4]. It can also be a subclinical condition [4–6]. Symptoms often overlap or are confused with gastrointestinal symptoms or lower pelvic/gynecologic symptoms, and diagnosis of LC is often delayed. LC can present concomitantly with the kidney [8] and/or gastrointestinal involvement of SLE [10, 11]. Imaging and urodynamic studies reveal variable degrees of bladder wall thickening, decreased capacity, urinary tract dilation, and smooth muscle contractibility [12, 13]. Histologically, leukocyte mononuclear infiltrate, haemorrhage, and the variable presence of fibrosis are found; deposits of immunoglobulins (IgA, IgG, and IgM) and complements (C1q and C3) can be identified around the small vessel walls of the bladder [14, 15]. Also observed is a variable degree of infiltration by mast cells, which could play a pathogenic role [16, 17]. Different therapeutic tools have been useful for disease control, including glucocorticoids [18, 19], antihistamines [9], cyclophosphamide [20], azathioprine [20], mycophenolate mofetil [10], intravenous immunoglobulin, and plasmapheresis [21]. This report presents four cases of Colombian patients with SLE and a noninfectious very severe and refractory form of cystitis (VSRC) that showed no response to conventional treatments. The majority of studies are from oriental populations, and reports are scarce in the Western world. A description is provided of its clinical and therapeutic characteristics and of possible factors related to this atypical and devastating complication.

## 2. Case Report

In a cohort of 240 patients with SLE treated since 2007 at the Fundación Valle del Lili, a reference centre for patients in southwestern Colombia, 12 (5%) had clinical, laboratory, cystoscopic, or histological manifestations consistent with LC at some points, which improved rapidly with conventional treatment. Of these, four patients developed VSRC, for which the clinical characteristics of the underlying disease, form of presentation, laboratory, imaging, cystoscopic, and histological findings, established treatments, and outcomes are described.

### 2.1. Case 1

Case 1 involves a 45-year-old woman with a diagnosis of SLE from the age of 40 who initially presented skin conditions (malar erythema and alopecia areata) and then Libman–Sacks endocarditis, polyarthritis, dryness symptoms (xerostomia and xerophthalmia), and hypothyroidism. She showed positivity for several autoantibodies, including antinuclear antibody (ANA) titres of 1/1280 with a speckled pattern, anti-Sm antibodies, anti-U1 RNP antibodies, direct Coombs, both IgM and IgG anticardiolipin antibodies, and complement consumption. She initially received glucocorticoids in high doses, which were reduced until they were discontinued two years later due to disease remission. A year later, the patient presented disease relapsing, and this time, she became refractory to glucocorticoids, hydroxychloroquine, and azathioprine; therefore, mycophenolate mofetil was indicated, without improvement; it was decided to start a rituximab cycle of 1 g on day 0 and 1 g on day 14, with a very favourable response; it was decided to maintain rituximab in a similar cycle every nine months for three years as monotherapy. One year after this treatment, glucocorticoids were discontinued. At 44 years old, 4 years after the onset of SLE and two years after entering remission and receiving rituximab monotherapy, she began to present with polyuria, dysuria, and hypogastric pain. Urinalysis shows leukocyturia without proteinuria; a urine culture was negative; a haemogram showed mild leukopenia; and haemoglobin, normal platelets, C-reactive protein (CRP), erythrocyte sedimentation rate (ESR), and C3 and C4 levels were normal. A cystoscopy was performed that showed urethral narrowing and inflammatory changes in the bladder wall. A bladder biopsy was performed, showing mucosal ulceration, lymphoplasmacytic infiltrate, and a slight presence of mast cells, without demonstrating deposits of viral particles in the immunohistochemistry nor deposits of immunoglobulins or complement fractions. Treatment was started with fexofenadine, 180 mg day, montelukast, 10 mg day, and amitriptyline, 25 mg day, without improvement. Symptoms worsened further; therefore, intravenous methylprednisolone was initiated at a rate of 500 mg/day for three days and continued at 1 mg/kg orally. In the absence of a response, azathioprine was added at 2 mg/kg. Rituximab was discontinued at the onset of urinary symptoms. There was slow progression towards improvement. Six months later, she continued with antihistamine, azathioprine, and methylprednisolone, 4 mg daily, and amitriptyline, 50 mg daily. Dysuria, nocturia, and mild polyuria persisted. Currently, SLE is in remission, and urinalysis showed moderate leukocyturia. Urine cultures for bacteria, mycobacteria, and fungi are consistently negative.

### 2.2. Case 2

Case 2 involves a 23-year-old woman with a diagnosis of SLE since 14 years of age who initially presented with adynamia, fever, headache, severe polyarthritis, class IV lupus nephritis, inflamed lung tissue (pleuritis and pneumonitis), anaemia, thrombocytopenia, Raynaud's phenomenon, and ANA titres of 1 : 1280, with a homogeneous pattern. Initially, she was treated with high doses of glucocorticoids, intravenous cyclophosphamide, 750 mg, with three intravenous doses every month, and then continued with azathioprine, 2 mg per kg, with which no favourable response was observed. Three months later, mycophenolate mofetil, 3 g/day, was started, with an adequate initial response but a severe relapse nine months later, mainly at the musculoskeletal and renal levels. It was decided to start rituximab, 1 g on day 0 and 1 g on day 14, with a very favourable response, and it was decided to continue rituximab maintenance in a similar cycle every 9–12 months for five years. She required prednisolone for four years, during which the dose could be reduced, and then, the medication was discontinued. In the second year of the disease, the patient developed avascular necrosis in the left femoral head that required surgical decompression and then joint replacement. Seven years after the SLE diagnosis and after four years of clinical and paraclinical remission and monotherapy with rituximab, dysuria, nocturia, polyuria, perineal pain, bladder pressure, and vesical tenesmus began. Urinalysis showed leukocyturia without proteinuria with a negative urine culture. A haemogram showed mild microcytic anaemia and normal CRP, ESR, kidney function, and C3 and C4 levels. Tests were negative for anti-DNA antibodies. MRI of the abdomen showed thickening of the bladder walls and decreased bladder capacity, with bilateral symmetrical and moderate hydroureteronephrosis. There were no symptoms or abnormalities in gastrointestinal images. Cystoscopy showed diffuse erythema throughout, including ureteral orifices, without affecting the rest of the trigone. Based on a biopsy, severe acute and chronic ulcerated cystitis with moderate multifocal neovascularization was observed, and up to 12 mast cells per high-power field were identified. Immunohistochemistry revealed inflammatory cells with abundant CD45-positive lymphocytes with few CD20-positive B lymphocytes and abundant CD5-positive T lymphocytes and, on occasion, CD38- and CD138-positive plasmocytes and abundant CD68-positive macrophages. Symptomatic treatment was administered with analgesics, antispasmodics, amitriptyline, and prednisolone (1 mg/*k*/day) without a response; azathioprine (100 mg/day) was added, and then, mycophenolate mofetil (2 g/day) was added, without adequately controlling the symptoms. Rituximab was not indicated again. She then received oxybutynin, hydroxyzine, carbamazepine, and gabapentin, without improvement of pain or polyuria. Urodynamics were analysed, showing a reduced bladder capacity of 36 cc, and intravesical botulinum toxin was indicated, with progressive improvement of the symptoms. One year later, the patient remains asymptomatic, with normal urinalysis, with parameters indicating SLE remission and without medication.

### 2.3. Case 3

Case 3 involves a 24-year-old woman with an SLE diagnosis at 19 years of age who initially presented with grade IV lupus nephritis, bicytopenia (lymphopenia and thrombocytopenia), complement consumption, and positive ANA and anti-DNA antibody results. Initially, she received methylprednisolone in intravenous pulses of 500 mg for three days and then 1 mg per kg, hydroxychloroquine, 200 mg/day, and intravenous cyclophosphamide, 750 mg each dose for three months, and then continued with mycophenolate mofetil, 3 g/day. The dose of methylprednisolone was reduced as clinical improvement occurred and was discontinued after three years of use. Four years after the SLE diagnosis and one year after presenting clinical and paraclinical remission and maintenance with mycophenolate mofetil, 2 g/day, and hydroxychloroquine, 200 mg three times a week, she presented general symptoms, polyarthritis with reappearance of proteinuria (15 gr in 24 hours), leukocyturia, and marked elevation of anti-DNA antibodies; therefore, methylprednisolone was again added to the treatment, initially 1 mg/kg and then a 1 g rituximab infusion and another 1 g at 2 weeks. One month later, dysuria, polyuria, nocturia, vesical tenesmus, and hypogastric pain began. Urinalysis showed leukocyturia and haematuria, with no increase in proteinuria, which at that time was 1 g in 24 hours. Urine cultures for common germs, mycobacteria, and fungi were negative. The symptoms quickly worsened, requiring her admission to the hospital for treatment. This hospitalization lasted three months without satisfactory control of the symptoms. The criteria for lupus activity indicated remission. Her kidney function, blood count, CRP, and ESR remained normal, C3 and C4 levels were normal, and there was a decrease in the anti-DNA antibody titre. Cystoscopy showed substantial oedema of the trigone, meatus, and bladder walls, with petechiae, and some ulcers, with residual urine of 50%. A biopsy showed chronic and acute ulcerated cystitis with exuberant granulation tissue and up to 10 mastocytes per high-power field. Immunohistochemistry did not show deposits of immunoglobulin or complement fractions. No germs were found. Nuclear magnetic resonance imaging of the pelvis showed thickening of the bladder walls with inflammatory changes. During this hospitalization, she received symptomatic treatments with analgesics including opioids, antihistamines (hydroxyzine, loratadine, fexofenadine, and ranitidine), various nonsteroidal anti-inflammatory drugs, antispasmodics, duloxetine, glucocorticoids (methylprednisolone, 0.5 mg/kg) by infusion or orally, and amitriptyline and pregabalin in high doses. Therapy was continued with mycophenolate mofetil, and intravenous gamma globulin was indicated at a rate of 400 mg/kg/day for five consecutive days. Plasmapheresis was indicated. Serum tryptase levels were normal, and she did not present other clinical or laboratory criteria for the diagnosis of systemic mastocytosis. Polyuria, nocturia, dysuria, and perineal pain were severe and refractory, requiring additional pelvic floor therapies, epidural analgesia, pudendal nerve blocks, and periurethral triamcinolone infiltrations. The application of botulinum toxin was proposed, but there was no agreement between the doctors or acceptance by the patient. The patient was discharged with partial improvement. The last urinalysis remained unchanged. Equipment for neurostimulation and/or possible application of botulinum toxin in the bladder wall were indicated for outpatient treatment.

### 2.4. Case 4

Case 4 involves a 20-year-old woman diagnosed with SLE at 12 years of age, initially presenting with grade IV lupus nephritis, haematological compromise (pancytopenia and positive direct Coombs autoimmune haemolytic anaemia), mucocutaneous compromise (discoid lupus), polyarthritis, central nervous system compromise (convulsions), and multiple positive autoantibodies at high titres, including ANAs, anti-DNA antibodies, anti-Sm antibodies, anticardiolipins IgG and IgM, and severe complement consumption. She received treatment with high-dose glucocorticoids both intravenously (pulses of methylprednisolone on several occasions) and orally; hydroxychloroquine, azathioprine, and mycophenolate mofetil with which she presented refractoriness; and monthly intravenous cyclophosphamide during several periods of illness related to relapses, with which she presented an improved therapeutic response. Six years after a period of remission, relapse in the mucocutaneous, musculoskeletal, and renal components occurred. Rituximab, 1 g on day 0 and 1 g on day 14, was initiated, with a favourable response, and rituximab was continued as maintenance therapy in a similar cycle every 9 months. Glucocorticoids were lowered, and the application of cyclophosphamide was not necessary during this time. After the last application of rituximab, she presented with symptoms of dysuria, polyuria, and nocturia; cystoscopy was performed, showing mucosal detachment, and a biopsy revealed inflammation with lymphoplasmacytic infiltration, mucosal necrosis, and the presence of fibrin. Microorganisms were not pregnant. The symptoms were refractory; she received methylprednisolone, bladder instillations with lidocaine and heparin, oxybutynin, and montelukast, without improvement. Kidney failure developed rapidly, with creatinine elevated to 2.6 mg/dL, haematuria, proteinuria, lymphopenia, C3 consumption and rapid onset diarrhoea, leading to the diagnosis of associated ulcerative colitis. Kidney failure progressed to a requirement for haemodialysis and management in an intensive care unit, where she presented with multiple severe infectious complications (disseminated histoplasmosis, parotid abscess due to *S. aureus*, pneumonia and right empyema requiring decortication, lower limb erysipelas, hip and gluteal abscesses requiring drainage, bacteraemia, and septic arthritis by *P. aeruginosa*). The patient died with multisystem failure. Urinary symptoms throughout this period were present despite various symptomatic treatments, including opioid analgesics.

Table 1 presents the clinical manifestations and treatment of SLE in four women before the onset of VSRC, clinical and paraclinical manifestations of VSRC, treatments received, and final outcomes. SLE, systemic lupus erythematosus. VSRC, very severe and refractory cystitis. IV, intravenous.

## 3. Discussion

The present report describes the cases of four women with SLE who presented manifestations related to the disease that include variable mucocutaneous, musculoskeletal, renal, haematological, neurological, and immunological involvement associated with the presence of autoantibodies (ANAs, anti-DNA antibodies, and anti-Sm antibodies) and complement consumption (C3 and C4), which were managed with conventional treatments such as glucocorticoids, azathioprine, intravenous cyclophosphamide, and mycophenolate mofetil with partial clinical response; therefore, it was necessary to add rituximab to their treatment plan, as described [22]. With this medication, three of these patients showed initial clinical and paraclinical improvement; therefore, maintenance therapy continued in the form of cycles approximately every nine months. The last patient received only the first cycle of rituximab. Three patients presented disease remission, and while under maintenance treatment with rituximab, they developed manifestations of VSRC. Due to its unusual presentation, VSRC is not classified as conventional LC, and we believe that the name VSRC best describes this disease.

The severity of presentation of this unusual cystitis with a chronic evolution manifested by severe dysuria, nocturia, polyuria, and perineal pain is striking, making it a clinically devastating condition. The therapeutic efforts described were quite varied, and refractoriness was consistent. The patients required extended hospitalizations, with death as an outcome in one patient who developed severe reactivation of SLE as well as opportunistic infections and multisystemic failure. This patient was part of a mortality analysis study [23].

Pathology analyses were consistent with severe forms of interstitial cystitis [24], ruling out the presence of neoplasms or infectious agents.

The presence of SLE remission in three of the patients at the time of VSRC onset and while receiving rituximab maintenance treatment is quite striking. It can be concluded that VSRC is refractory to this treatment. What cannot be concluded is whether rituximab is part of the pathogenesis of this type of condition. To date, no association has been described between rituximab and refractory cystitis of noninfectious aetiology. Several long-term adverse reactions have been implicated with rituximab, including hypogammaglobulinemia associated with serious infections [25], arrhythmias such as supraventricular tachycardia [26], bowel obstruction and perforation associated with lymphoproliferative disorders [27], skin disorders such as pemphigus, lichenoid, or vesiculobullous eruptions up to 3 months after infusions [28], vulvovaginal pyoderma gangrenosum [29], and pulmonary complications such as interstitial lung disease or bronchiectasis [26, 30]. The development of bronchiectasis is related to being predisposed to infections and injury to the structures of the bronchial wall, if the tissues fail to be repaired. Extrapolating this manifestation to the VSRC that we are describing is difficult, but there is concern that there is an association.

We present four Colombia patients with SLE (three in remission and one with severe disease relapse) whose treatment was based on maintenance rituximab in three of the cases and onset in one of them, who presented a very severe form of cystitis refractory to different forms of treatment, including immunosuppression. Because LC is observed more frequently in Eastern ethnic groups and that in Colombia, there is a high prevalence of mestizo and mulatto populations, and it can be thought that some genetic factors are derived from heredity intervenes in the pathogenesis of this condition. More studies are needed related to this devastating pathology to better understand its pathogenesis and treatment as well as to clarify whether rituximab therapy plays a pathogenic role.

## Figures and Tables

**Table 1:** The clinical manifestations and treatment of SLE before the onset of VSRC, the clinical and paraclinical manifestations of VSRC, the treatments received, and the final outcome.

Case	Age at diagnosis of SLE (years)	Main manifestations of SLE	Treatments for inducing SLE remission	Maintenance treatment for SLE	Age at onset of VSRC (years)	SLEDAI 2 K at VSRC onset	Manifestations of VSRC	Treatments for VSRC with failed response	VSRC outcome, relationship with SLE activity
1	40	Mucocutaneous Musculoskeletal Cardiovascular (endocarditis) Immunological	Glucocorticoids Azathioprine Mycophenolate Mofetil Rituximab	Rituximab (3 cycles)	44	1	Dysuria Polyuria Nocturia	Methylprednisolone Azathioprine Fexofenadine Amitriptyline	Improvement, SLE inactive

2	14	Musculoskeletal Renal Pulmonary Haematologic Immunological	Glucocorticoids Mycophenolate Mofetil Cyclophosphamide Rituximab	Rituximab (6 cycles)	21	2	Dysuria Polyuria Nocturia Perineal pain Bladder pressure	Prednisolone Amitriptyline Hydroxyzine Oxybutynin Carbamazepine Gabapentin Botulinum toxin	Improvement, SLE inactive

3	19	Renal Musculoskeletal Haematologic Immunological	Glucocorticoids Mycophenolate Mofetil Cyclophosphamide	Mycophenolate Hydroxychloroquine Rituximab started at relapse (one cycle)	23	8	Dysuria Polyuria Severe perineal pain Bladder pressure Hypogastric pain	Methylprednisolone Amitriptyline Anti-H1 Anti-H2 : ranitidine NSAIDs Opioids Pregabalin Hyoscine bromide Intravenous gamma globulin Pudendal block Urethral infiltration (partial response)	Refractoriness to treatment, SLE inactive

4	12	Renal Haematologic Immunological Mucocutaneous Neurological Gastrointestinal	Glucocorticoids Azathioprine Mycophenolate Mofetil Cyclophosphamide Rituximab	Rituximab (7 cycles)	20	12	Dysuria Polyuria Nocturia	Methylprednisolone IV Instillations (heparin and lidocaine) Oxybutynin Montelukast	Refractory treatment, relapse of SLE. Death associated with infections, multisystem failure.

## Data Availability

No data were used to support this study.
